# The complete mitochondrial genome and phylogenetic position of the leopard catshark, *Poroderma pantherinum*

**DOI:** 10.1080/23802359.2018.1483772

**Published:** 2018-07-10

**Authors:** Michaela van Staden, Katie S. Gledhill, Clint Rhode, Aletta E. Bester-van der Merwe

**Affiliations:** a Department of Genetics, Stellenbosch University, Stellenbosch, South Africa;; b South African Shark Conservancy, Old Harbour Museum, Hermanus, South Africa

**Keywords:** mitochondrial genome, catshark, *Poroderma pantherinum*, Scyliorhinidae

## Abstract

We present the first mitochondrial genome of a South African endemic catshark, *Poroderma pantherinum*. The complete mitogenome is 16,686 bp in length, comprising 13 protein-coding genes, 2 rRNA genes, 22 tRNA genes, and one non-coding control region. Similar to other shark mitogenomes, it is AT rich (61.1%), with a GC content of 38.9%. Protein-coding genes used one of two start codons (ATG and GTG) and one stop codon (TAA/TA-/T-). Phylogenetic analysis of the leopard catshark and 34 carcharhinid species showed that it clusters with two other scyliorhinid species (*Cephaloscyllium umbratile* and *Scyliorhinus canicula*) with 100% support.

The leopard catshark *Poroderma pantherinum* (Carcharhiniformes: Scyliorhinidae) is a bottom-dwelling South African endemic shark, predominantly distributed in inshore waters along the south and south-east coasts (Human 2006). There is a lack of data on the population trends of *P. pantherinum* and it is currently assessed as Data Deficient using the International Union for the Conservation of Nature (IUCN) Red List Criteria (Human 2009). Southern Africa is a biodiversity hotspot with many endemic catsharks (Ebert and van Hees 2015); however, the lack of genetic resources for South African sharks delays the understanding of species delineation, population genetics, and reproductive behaviour (Bester-van der Merwe and Gledhill 2015). Here, we present the first complete mitogenome sequence and phylogenetic position of a South African endemic catshark.

A tissue sample (fin clip) was taken from one female *P. pantherinum* individual in Walker Bay, Hermanus, South Africa (geospatial coordinates: –34.421111, 19.244010) in 2016. The fin clip sample (FWB387) is stored in 100% ethanol at Stellenbosch University, Department of Genetics. Total genomic DNA was isolated using a standard cetyltrimethylammonium bromide (CTAB) extraction protocol (Sambrook and Russell 2001). Low coverage whole genome sequencing was performed on an Ion Torrent S5™ platform (Thermo Fisher Scientific, Waltham, MA, USA). Libraries with a mean insert size of 600 bp were prepared for sequencing using the Ion Plus Fragment Library Kit (Thermo Fisher Scentific), following the manufacturer’s protocol. The generated sequence reads were quality filtered using Torrent Suite™ Software (Thermo Fisher Scientific) and mapped to a reference mitogenome from *Scyliorhinus canicula* (NC_001950.1) in Geneious® v.10.2.3 (Kearse et al. 2012). The final assembly was annotated using MitoAnnotator (Iwasaki et al. 2013). A MUSCLE alignment, excluding *ND6* and the control region, was performed in Geneious^®^ with 36 publicly available elasmobranch mitogenomes. A Bayesian tree (Figure 1) was generated in MrBayes v.3.2.6 (Huelsenbeck and Ronquist 2001) using the best substitution model (GTR + I + G) determined by the Bayesian Information Criterion in jModelTest2 v.0.1.10 (Darriba et al. 2012). The Bayesian analysis was run for 1,000,000 generations, sampled every 1000 generations and the first 25% of trees were omitted as burn-in with the remaining trees used to calculate the posterior probabilities.

**Figure 1.**
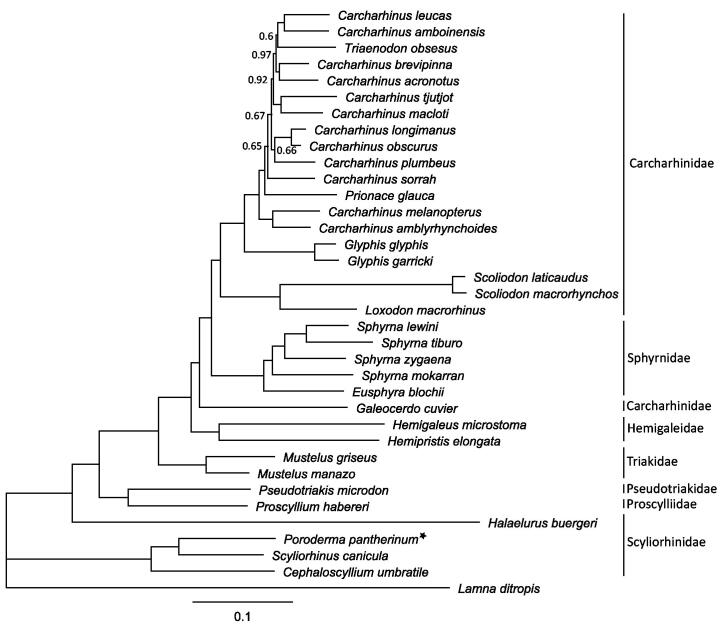
Bayesian tree depicting the phylogenetic position of *Poroderma pantherinum* (posterior probability values only shown if below 1.0). Based on 35 mitochondrial genomes (excluding *ND6* and the control region) of sharks from the order Carcharhiniformes, using *Lamna ditropis* (KF962053.1) as an outgroup. (*Carcharhinus acronotus*: NC_024055.1, *Carcharhinus amblyrhynchoides*: NC_023948.1, *Carcharhinus amboinensis*: NC_026696.1, *Carcharhinus brevipinna*: KM244770.1, *Carcharhinus leucas*: KF646785.1, *Carcharhinus longimanus*: NC_025520.1, *Carcharhinus macloti*: NC_024862.1, *Carcharhinus melanopterus*: NC_024284.1, *Carcharhinus obscurus*: NC_020611.1, *Carcharhinus plumbeus*: NC_024596.1, *Carcharhinus sorrah*: NC_023521.1, *Carcharhinus tjutjot*: KP091436.1, *Cephaloscyllium umbratile*: NC_029399.1, *Eusphyra blochii*: NC_031812.1, *Galeocerdo cuvier*: NC_022193.1, *Glyphis garricki*: KF646786.1, *Glyphis glyphis*: NC_021768.2, *Halaelurus buergeri*: NC_0311811.1, *Hemigaleus microstoma*: KT003687.1, *Hemipristis elongata*: KU508621.1, *Loxodon macrorhinus*: KT347599.1, *Mustelus griseus*: NC_023527.1, *Mustelus manazo*: NC_000890.1, *Prionace glauca*: NC_022819.1, *Proscyllium habereri*: KU721838.1, *Pseudotriakis microdon*: NC_022735.1, *Scoliodon laticaudus*: KP336547.1, *Scoliodon macrorhynchos*: NC_018052.1, *Scyliorhinus canicula*: NC_001950.1, *Sphyrna lewini*: NC_022679.1, *Sphyrna mokarran*: KY464952.1, *Sphyrna tiburo*: KM453976.1, *Sphyrna zygaena*: NC_025778.1 and *Triaenodon obsesus*: KJ748376.1).

The complete mitogenome of the leopard catshark (accession MH321446) is 16,686 bp in length, containing 13 protein-coding, 22 tRNA, 2 rRNA genes, and one non-coding control region. The nucleotide base composition is rich in A (31.0%) + T (30.1%) and low in C (25.0%) + G (13.9%), which is common for elasmobranch mitogenomes (Ruck et al. 2017). All genes started with the standard ATG codon, except *COI*, which started with the alternate GTG codon. Majority of the genes ended with the TAA stop codon, with *ND2*, *COII*, *ND3*, *ND4,* and *CYTB* ending with an incomplete stop codon (TA–/T–). The Bayesian tree (Figure 1) shows that *P. pantherinum* clusters with two other catshark species (*Cephaloscyllium umbratile* and *Scyliorhinus canicula*) with 100% support, whereas *Halaelurus buergeri* clusters with the remaining five families supporting earlier work by Chen et al. (2016) that the family Scyliorhinidae is paraphyletic.
